# The Costs of Home Monitoring by Telemedicine vs Standard Care for Inflammatory Bowel Diseases—A Danish Register-Based, 5-Year Follow-up Study

**DOI:** 10.1093/ecco-jcc/jjae120

**Published:** 2024-08-07

**Authors:** Marwah Al-Sheikh, Dorit Vedel Ankersen, Jens Olsen, Maria Spanggaard, Charlotte T Peters-Lehm, Rahim M Naimi, Mette Bennedsen, Johan Burisch, Pia Munkholm

**Affiliations:** Department of Gastroenterology, Medical Division, Copenhagen University Hospital, North Zealand, Denmark; Department of Gastroenterology, Medical Division, Copenhagen University Hospital, North Zealand, Denmark; b EY, Copenhagen, Denmark; b EY, Copenhagen, Denmark; Department of Gastroenterology, Medical Division, Copenhagen University Hospital, North Zealand, Denmark; Department of Gastroenterology, Medical Division, Copenhagen University Hospital, North Zealand, Denmark; Department of Gastroenterology, Medical Division, Copenhagen University Hospital, North Zealand, Denmark; Gastro Unit, Medical Division, Copenhagen University Hospital – Amager and Hvidovre, Hvidovre, Denmark; Department of Gastroenterology, Medical Division, Copenhagen University Hospital, North Zealand, Denmark

**Keywords:** Telemedicine, inflammatory bowel disease (IBD), cost-effectiveness

## Abstract

**Background and Aims:**

There are few studies on the cost-effectiveness of telemedicine for inflammatory bowel diseases. We assessed the long-term cost-effectiveness of a telemedicine solution compared to standard care (sCare), as well as its efficacy according to patient-reported outcomes (PROs).

**Methods:**

Between 2015 and 2020, we conducted a retrospective, register-based study among patients with ulcerative colitis and Crohn’s disease. Direct and indirect healthcare costs over a 5-year period were obtained from Danish registers and compared to a control group. Costs were estimated on a yearly basis from 1 year before, until 5 years after, inclusion in the trial. Patients were divided into 2 groups: those not receiving biologics (Cohort 1) and those receiving biologics (Cohort 2).

**Results:**

We recruited 574 patients with inflammatory bowel diseases. In Cohort 1 (61.5%), average total direct costs and total earnings per patient per year were €14 043 and €307 793, respectively, in telemedicine compared to €16 226 and €252 166, respectively, in sCare. In Cohort 2 (38.5%), average total direct costs and total earnings were €73 916 and €215 833, respectively, in telemedicine compared to €41 748 and €203 667, respectively, in sCare. PROs showed improved quality of life, which was higher in Cohort 1 than in Cohort 2. Disease activity among patients with Crohn’s disease increased after Years 3 and 4 in Cohorts 1 and 2, respectively.

**Conclusion:**

Telemedicine is cost-effective for patients not receiving biologics. However, treatment with biologics is more expensive for patients enrolled in telemedicine. Careful attention to PROs in telemedicine improves quality of life and could prolong the time to relapse.

## 1. Introduction

Inflammatory bowel diseases (IBDs), comprising Crohn’s disease (CD) and ulcerative colitis (UC), are chronic, relapsing, and remitting diseases of the gastrointestinal tract. IBDs are incurable diseases that require tailored, long-term medical treatment and close monitoring to maintain remission, as well as to ensure early detection of flare-ups and the prevention of possible complications such as surgery and reduced quality of life (QoL).^1–3^ Close monitoring following a treat-to-target strategy includes measurement of disease activity every 3 months in patients with active disease and every 6–12 months for patients in remission.^4^

IBDs are expensive diseases^5^ that are associated with high healthcare costs even in the years prior to their diagnosis.^6^ The anticipated increase in IBD prevalence^7,8^ and the growing use of expensive biologics and small molecules^9^ will place an increasing burden on the health sectors and society. A recent report about the economic burden imposed by IBDs in high-income settings found that IBD costs are substantial and primarily driven by the direct expenses of medical care, including hospitalizations, medications, and surgeries.^5^ These expenses were shown to escalate significantly as the disease progressed and worsened. The indirect costs of IBDs, such as lost productivity and sick leave, accounted for a significant portion of the disease’s overall economic burden.

Telemedicine is used worldwide as a communication tool between patients and healthcare professionals to improve disease management, adherence to treatment, and QoL. Studies have shown that telemedicine allows for the close follow-up of patients with chronic diseases such as IBDs and can improve their QoL, while reducing healthcare costs.^10,11^ Furthermore, the involvement of patients in their own care and in self-monitoring via telemedicine reduces the number of clinic visits and hospital admissions, as well as improves their QoL and health outcomes.^12^

Several telemedicine interventions have been developed to provide better care and lower direct and indirect healthcare costs for IBDs. These include MyIBDCoach in the Netherlands, TECCUS in Spain, TELE-IBD in the United States, and IBD-Home in Sweden.^13–16^ In Denmark, the patient-reported outcome (PRO)-based telemedicine solution, Constant Care (CC), has been used in our institution for remote monitoring of patients with IBD.^17–19^

To date, none of these interventions have been evaluated for their long-term efficacy in terms of PROs or cost-effectiveness. In this study, we aimed to evaluate the long-term cost-effectiveness of telemedicine solution compared to standard care (sCare) for IBDs over 5 years of follow-up. Secondarily, we aimed to explore the efficacy of telemedicine solution by investigating PROs within the telemedicine group.

## 2. Methods

### 2.1. Study design and web application

The study was designed as a retrospective 5-year follow-up register study to evaluate the direct and indirect costs per patient associated with using the telemedicine solution, CC. Nationwide Danish registers were used to match cases enrolled in CC with IBD controls and to retrieve healthcare costs and utilization rates.

### 2.2. Constant care

The telemedicine solution CC for the remote management of IBDs has been described in more detail elsewhere.^1,20^ Briefly, CC is a home-monitoring web application (https://ibd.constant-care.com/) developed in 2005 in Denmark and implemented in clinical practice in North Zealand Hospital (NOH) in 2015. The web application captures PROs such as disease activity, based on the Simple Clinical Colitis Activity Index (SCCAI) for UC and the Harvey–Bradshaw Index (HBI) for CD, and allows for home measurement of fecal calprotectin using CalproSmart (Calpro AS, Oslo, Norway). Using an inbuilt algorithm, CC calculates a Total Inflammatory Burden Score (TIBS) based on clinical disease activity and fecal calprotectin, which is then presented to the healthcare provider and to the patient in a traffic light system, where red indicates severe activity, yellow indicates mild-to-moderate activity, and green indicates remission.^21^ Based on the TIBS, monitoring and/or treatment plans are then adjusted. Patients using CC were advised to register their symptoms every other week, or every 2 months if they were asymptomatic.

**Figure 1:**
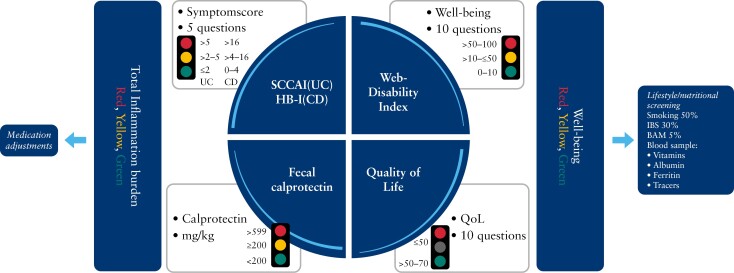
Overview of the screening principles of the telemedicine solution Constant Care. The left side of the shield is used for monitoring disease activity (total inflammation burden) and the right side for monitoring well-being. These home monitoring measures enable timely clinical decision-making. Abbreviations: BAM, bile acid malabsorption; CD, Crohn’s disease; HBI, Harvey–Bradshaw Index; IBS, irritable bowel syndrome; QoL, quality of life; SCCAI, Simple Clinical Colitis Activity Index; UC, ulcerative colitis.^22^ Adapted from Ankersen et al^22^; Copyright: permission is granted from John Wiley and Sons.

Beginning in 2015, the use of telemedicine has been recorded in the Danish National Patient Registry (NPR) with a specific code (procedure code ZPW00900) every time a patient has an interaction with a healthcare provider that results in a change to their treatment (Figure 1).

### 2.3. Patient populations

The case population comprised IBD patients aged 18 years or older who were enrolled in CC between July 8, 2015 and December 31, 2020 and treated at NOH. The index date was defined as the date of enrollment in CC. To ensure at least 1 year of follow-up, patients enrolled in CC in 2021 and 2022 were excluded from the study.

Patients with a primary diagnosis of CD or UC according to the *International Classification of Diseases*, 10th edition (ICD-10 codes DK50 and DK51, respectively), who were registered in the Danish NPR, but who were treated somewhere other than NOH, were identified as potential controls. Cases and potential controls were excluded if they had undergone a total colectomy before their index date.

Cases enrolled in CC were identified through a population file maintained by NOH. Data were combined with those from the Danish national registers and were cross-checked with the NPR to ensure that all cases had been diagnosed with UC or CD, treated at NOH, and were present in the Danish registers. Cases were matched with up to 5 controls identified in the NPR who were receiving sCare for their IBD. Cases and controls were matched based on the following variables: year of birth, sex, educational level, IBDs (CD or UC), IBD duration,^23^ and type and number of biologics (infliximab, adalimumab, golimumab, vedolizumab, ustekinumab, or tofacitinib) received in the 18 months preceding, and up to 6 months after, enrollment in CC. Danish regulations stipulate that biologics only can be provided at hospitals’ outpatient infusion units.^24^

Using registry data on the individual level, we followed study participants from 1 year before their enrollment in CC (ie, the index date) to up to 5 years after their index date. Participants were followed until their death, emigration, or the end of follow-up (December 31, 2020 for the cost analyses and December 31, 2021 for the healthcare utilization analyses).

Procedure codes are listed in Supplementary Figure 1 and its corresponding Supplementary Table 1.

### 2.4. Registries, outcome variables, and unit costs

Outcomes for this study included direct and indirect costs for cases compared to their matched controls, as well as the respective rates of healthcare utilization.

Direct costs included the healthcare costs in the primary healthcare sector and the hospital care sector (outpatient and/or inpatient, including diagnostic procedures), as well as costs related to prescription medicines and home care services. Primary healthcare sector costs were estimated based on fees for primary healthcare services retrieved from the Danish National Health Service Register for Primary Care.^25^ Costs in the hospital care sector included hospital admissions and outpatient visits at the hospital and were estimated using Diagnosis-Related Group (DRG) tariffs retrieved from the NPR.^23^ DRG tariffs for outpatient costs also include the costs of biologics.

The NPR is a comprehensive database of all hospital admissions and outpatient visits in Denmark since 1977. It serves as a valuable resource for medical research, health policy evaluation, and epidemiological studies. The registry contains detailed data on patient demographics, diagnoses, treatments, and outcomes, making it a reliable source of information for studying disease patterns, healthcare utilization, and treatment effectiveness.^23^

Denmark provides partial reimbursement of prescribed medications via the Central Reimbursement Register (CTR). However, immunomodulators such as methotrexate and azathioprine, as well as biologics, are provided to patients for free and are paid for by Denmark’s income tax. These drugs are provided at the hospital departments or at pharmacy personal lockers.^26,27^ Product pricing is determined through negotiations by the Danish Medicines Council.^28^

We used the NPR to estimate the number of endoscopic procedures and radiological procedures, including computer tomography scans and magnetic resonance imaging, carried out on patients. We also estimated the number of IBD-related surgeries, defined as bowel resections, total colectomies, and resections and excisions of the rectum.

Both hospital admission costs and outpatient visit costs were stratified as IBD-related and non-IBD–related costs. IBD-related visits were defined as visits where the primary or secondary diagnosis code was CD or UC. Prescription medicine costs were calculated using the pharmacy prices (including the Danish value-added tax of 25%) and according to Danish reimbursement policy.^29^ The costs of prescription medicines were retrieved from the NPR, which includes records of all fulfillments by Danish pharmacies. Home care service costs were estimated using the hours of nursing and practical services recorded in the Register of Municipal Services multiplied by the average hourly rate for healthcare workers (€33.34), with an overhead of 100%, per Danish guidelines.^30–32^

The indirect costs of possible losses in productivity were defined as the difference in earnings between cases and their controls. Data about income were retrieved from the Income Statistics Register.^33^

All costs and earnings were inflated to 2021 prices according to the consumer price index and converted to Euros (DKK 7.5 = EUR 1).

Healthcare utilization rates were estimated as the number of hospital admissions and outpatient visits and were stratified by IBD-related and non-IBD–related contacts, as well as the number of primary care visits.

Further, differences in the proportions of early retirement and public transfer payments were estimated using data from the DREAM (Danish: *Den Registerbaserede Evaluering Af Marginaliseringsomfanget*) database.

We included the PRO scores recorded in the CC for cases as secondary endpoints. These included QoL (measured by the Short Inflammatory Bowel Disease Questionnaire [SIBDQ]),^34^ SCCAI,^35^ HBI,^36^ Web Disability Index (WEB-DI),^37^ Medication Adherence Rating Scale (MARS),^38^ as well as fecal calprotectin measured at home. These data were obtained only for cases in CC and were stratified according to whether they were not using biologics, that is, only using 5-aminosalicylic acid (5-ASA), azathioprine, and methotrexate (Cohort 1), or were using biologics (Cohort 2). All data were imported into patients’ EPIC files.

### 2.5. Statistical analyses

Using register data from 2013 onward, the yearly average healthcare utilization rates and costs from 1 year before, until 5 years after, the index date were estimated for cases and controls for each cost component, that is, primary healthcare costs, hospital admission costs, outpatient visit costs, prescription medicine costs, home care costs, and earnings. Hospital utilization, hospital admission costs, and outpatient visit costs were also stratified by IBD-related and non-IBD–related costs and contacts. A linear regression model was used to determine statistically significant differences in costs between cases and controls. Likewise, we approximated differences in indirect costs between cases and controls by comparing yearly average earnings and estimated statistically significant differences using a linear regression model.

We used PROs from CC to estimate the yearly average QoL, SCCAI score, HBI score, WEB-DI score, MARS score, and fecal calprotectin from the index date until 5 years after the index date. As cases were allowed to answer the questionnaires several times per year, in order to calculate the average scores, all patients were weighted equally.

All analyses were conducted for Cohort 1 (cases not receiving biologics in the study period and their respective matched controls) and Cohort 2 (cases receiving biologics anytime during the study period and their respective matched controls). Cases needed to have received a minimum of 3 treatments with biologics in the 18 months prior to their index date in order to be included in Cohort 2. Cases receiving 3 treatments with biologics after the index date were also included in Cohort 2.

Analyses of PROs were conducted only with cases in CC, as PROs are not recorded in the Danish registers.

Data management and statistical analyses were carried out using *R* statistical software (version 3.4.4) on Statistics Denmark’s research computers via a remote server.

### 2.6. Ethical considerations

This register-based study complies with the regulations and instructions set forth by Statistics Denmark. We used only anonymized data and present these data here anonymously and only in aggregate form. We neither contacted nor required any active participation from any study participants. Ethics Committee approval and written informed consent are not required for register-based research according to Danish law. The study was approved by the Capital Region’s Knowledge Center for Data Reviews, journal-nr: P-2022-530. Permission from the Danish Data Protection Authority is no longer a requirement for studies such as this one.

## 3. Results

### 3.1. Study population

Between 2015 and 2022, 1000 IBD patients were enrolled in CC. Of those, 410 patients were excluded due to too short a follow-up time, 8 patients were excluded because of a lack of data, and 7 patients were excluded due to having undergone a total colectomy. One case was excluded because they could not be matched with a control. In total, 574 cases enrolled in CC between 2015 and 2020 were included in the analyses. Of those, 353 cases (61.5%) were not treated with biologics during the study period (Cohort 1). A total of 221 cases (38.5%) were included in Cohort 2 as they had received biologics at some time during the study period; 178 cases had received biologics in the 18 months preceding the index date and 43 cases had received biologics after the index date, while 966 controls (35%) had received biologics at some point in the study period. Of interest is the fact that the use of biologics (infliximab, vedolizumab, ustekinumab, and tofacitinib) was higher among cases than controls following their index dates (Table 1).

**Table 1:** Characteristics of cases in CC and their matched controls receiving sCare.

	Case in CC telemedicine	Control in sCare
*N*, total	574	2.740
Cohorts		
Cohort 1^a^	353 (61.5%)	1695 (61.9%)
Cohort 2^a^	221 (38.5%)	1045 (38.1%)
Type of disease		
CD^a^	235 (41%)	1141 (42%)
UC^a^	339 (59%)	1599 (58%)
Year of inclusion		
2015	56 (10%)	
2016	34 (6%)	
2017	0 (0%)	
2018	81 (14%)	
2019	263 (46%)	
2020	140 (24%)	
Years since IBD diagnosis, median (IQR)	5 (1, 11)	6 (3, 11)
Age, median (IQR)	44 (33, 54)	44 (33, 53)
Gender		
Male	249 (43%)	1178 (43%)
Female	325 (57%)	1562 (57%)
Use of biologics 18 mo before index date	178 (31%)	840 (31%)
Use of biologics in the full study period	221 (39%)	966 (35%)
Type of biologics used in the 18 mo before index^a^		
Infliximab	104 (18%)	438 (16%)
Adalimumab	37 (6%)	177 (6%)
Golimumab	<5 (<1%)	51 (2%)
Vedolizumab	54 (9%)	230 (8%)
Ustekinumab	18 (3%)	78 (3%)
Tofacitinib	NA (0%)	<5 (0%)
Type of biologics used in the study period (1 y before to 5 y after)		
Infliximab	144 (25%)	539 (20%)
Adalimumab	64 (11%)	317 (12%)
Golimumab	9 (2%)	79 (3%)
Vedolizumab	95 (17%)	311 (11%)
Ustekinumab	42 (7%)	163 (6%)
Tofacitinib	6 (1%)	9 (0%)

Abbreviations: CC, Constant Care; CD, Crohn’s disease; IBD, inflammatory bowel disease; IQR, interquartile range; NA, not available; sCare, standard care; UC, ulcerative colitis.

a Cohort 1: Cases not receiving biologics; Cohort 2: Cases receiving biologics.

### 3.2. Healthcare utilization

Healthcare utilization rates are shown in Table 2.

**Table 2.** Mean number of hospital admissions and outpatient visits, stratified by IBD-related and non-IBD–related contacts, as well as the number of primary care visits per patient per year, from 1 y before, until 5 y after, the index date for cases in Constant Care and matched controls in standard care for Cohort 1 (cases not receiving biologics) and Cohort 2 (cases receiving biologics).

	Year −1 (baseline)			Year 1 (index)			Year 2			Year 3			Year 4			Year 5		
	Case	Control	*p* Value	Case	Control	*p* Value	Case	Control	*p* Value	Case	Control	*p* Value	Case	Control	*p* Value	Case	Control	*p* Value
Cohort 1, *N*	351	1.698		302	1.438		181	875		68	329		68	319		60	282	
Primary healthcare visits	12.47	12.49	0.981	10.53	11.46	0.116	9.25	9.09	0.838	13.35	14.12	0.535	14.84	13.66	0.343	10.48	9.43	0.427
Outpatient visits:	6.39	4.01	0.000	5.56	4.57	0.000	4.78	4.13	0.011	4.61	3.37	0.000	3.76	3.46	0.496	5.31	4.84	0.419
IBD-related visits	3.86	1.36	0.000	3.22	1.52	0.000	2.73	1.30	0.000	2.05	1.05	0.000	1.36	0.98	0.091	2.32	1.27	0.000
Non-IBD–related visits	2.53	2.66	0.546	2.34	3.05	0.000	2.05	2.84	0.000	2.56	2.32	0.311	2.40	2.48	0.805	2.99	3.57	0.209
Hospital admissions:	0.46	0.40	0.165	0.29	0.28	0.706	0.23	0.24	0.902	0.22	0.22	0.957	0.18	0.17	0.961	0.21	0.20	0.937
IBD-related admissions	0.13	0.06	0.000	0.06	0.05	0.374	0.07	0.03	0.005	0.02	0.02	0.978	0.03	0.03	0.987	0.06	0.03	0.470
Non-IBD–related admissions	0.34	0.34	0.981	0.23	0.23	0.932	0.16	0.21	0.236	0.20	0.20	0.944	0.14	0.14	0.952	0.15	0.16	0.855
Cohort 2, *N*	221	1.044		190	884		94	425		22	96		22	96		20	86	
Primary healthcare visits	16.32	13.96	0.009	14.17	11.92	0.022	11.25	8.93	0.097	21.05	15.82	0.069	20.36	16.39	0.167	12.75	12.04	0.814
Outpatient visits:	15.07	11.75	0.000	14.50	11.22	0.000	13.20	9.36	0.000	9.79	6.60	0.000	8.88	5.37	0.000	11.68	8.31	0.023
IBD-related visits	11.10	7.81	0.000	10.63	7.16	0.000	9.88	5.54	0.000	6.97	3.54	0.000	5.16	2.88	0.001	8.27	3.79	0.000
Non-IBD–related visits	3.97	3.93	0.903	3.86	4.06	0.572	3.32	3.81	0.144	2.82	3.06	0.539	3.73	2.49	0.080	3.41	4.52	0.303
Hospital admissions:	0.82	0.70	0.141	0.44	0.41	0.751	0.27	0.35	0.282	0.29	0.26	0.701	0.59	0.24	0.037	0.64	0.32	0.213
IBD-related admissions	0.48	0.32	0.001	0.20	0.17	0.504	0.11	0.10	0.896	0.12	0.07	0.280	0.14	0.03	0.261	0.55	0.08	0.002
Non-IBD–related admissions	0.34	0.38	0.480	0.24	0.25	0.929	0.16	0.25	0.125	0.17	0.19	0.751	0.45	0.21	0.055	0.09	0.24	0.449

Abbreviation: IBD, inflammatory bowel disease.

In Cohort 1, there was no significant difference in primary healthcare visits between cases and controls. In Cohort 2, cases had more primary healthcare visits than their matched controls; however, this difference only reached statistical significance in Years −1 and 1 (*p* < 0.05).

Throughout the study period, cases had more outpatient visits than controls in both cohorts (Table 2), which was also true for IBD-related outpatient visits. In Cohort 1, cases had significantly fewer non-IBD–related outpatient visits in Years 1–3.

In Cohort 1, there were no significant differences in hospital admissions between cases and controls. In Cohort 2, cases had significantly more admissions than controls in Year −1 (*p* = 0.001) and Year 5 (*p* = 0.002).

### 3.3. Direct healthcare costs

**Figure 2:**
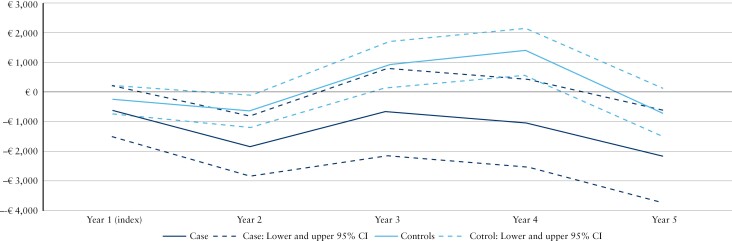
Costs incurred by Cohort 1 (ie, cases and their matched controls not receiving biologics) in the study period. Using Year −1 as a baseline, the figure shows the mean incremental total direct costs per patient per year from Years 1 to 5 after the index date for cases in Constant Care and their matched controls receiving standard care. Abbreviations: CI, confidence interval; IBD, inflammatory bowel disease.

**Figure 3:**
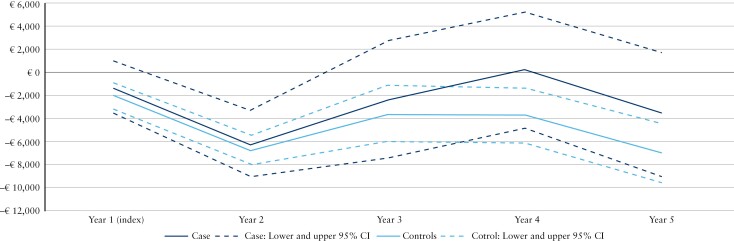
Costs incurred by Cohort 2 (ie, cases and their matched controls receiving biologics) in the study period. Using Year −1 as a baseline, the figure shows the mean incremental total direct costs per patient per year from Years 1 to 5 after the index date for cases in Constant Care and their matched controls receiving standard care. Abbreviations: CI, confidence interval; IBD, inflammatory bowel disease.

Using Year −1 as a baseline, Figures 2 and 3 present the mean incremental total direct costs per patient per year from Years 1 to 5 after the index date for cases in telemedicine and their matched controls in sCare for the 2 cohorts, respectively.

In Cohort 1 (Figure 2), the total direct healthcare costs were significantly higher for cases than controls from Year −1 to Year 1. Total direct healthcare costs trended downwards in Years 2–5, although the differences only reached statistical significance in Year 4. The main cost drivers among cases in Cohort 1 and their controls were outpatient visits, followed by prescription medicines and hospital admissions.

Cohort 2 generated higher direct healthcare costs overall than Cohort 1 (Figures 2 and 3), especially in the costs of outpatient visits (and including the costs of biologics). In Cohort 2 (Figure 3), the total direct healthcare costs were higher for cases than for controls both in Year −1 and after the index date (Years 1–5) (*p* < 0.001). The main cost drivers among cases in Cohort 2 and their controls were IBD-related outpatient visits. No difference was seen in the hospital admission costs between cases and controls, but IBD-related hospital admission costs trended downwards in Years 1–3, albeit with a significant increase in IBD-related admissions observed in Year 4.

The costs of prescription medicines for cases were higher than those for controls in both cohorts and across all years; however, this difference did not reach statistical significance in Year 3 for Cohort 2 and Year 4 for Cohort 1. In Cohort 2, there was a trend toward slightly lower costs for prescription medicines in Year 5.

**Figure 4:**
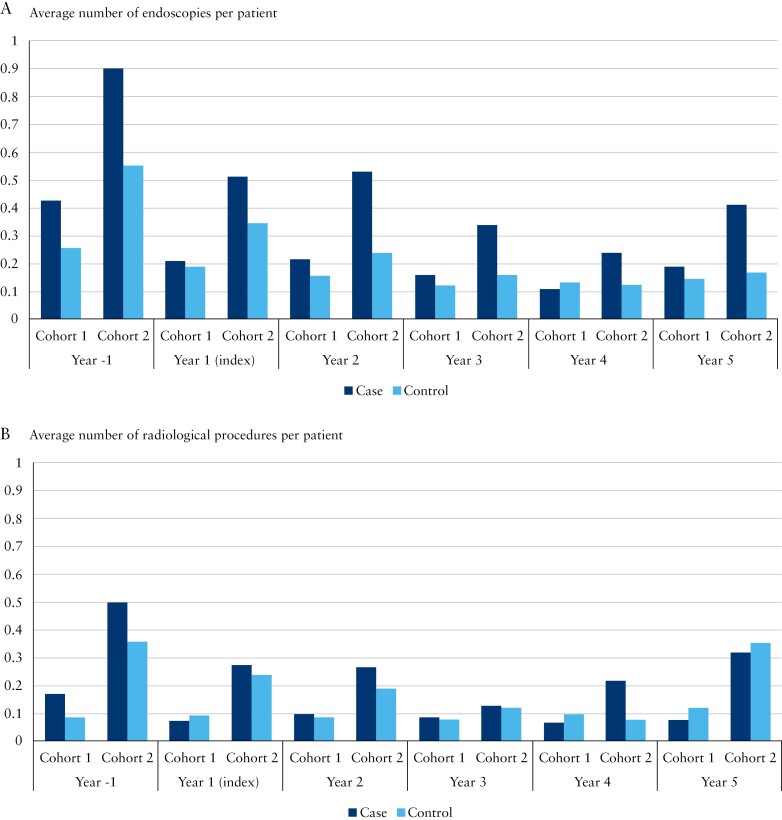
Mean number of (A) endoscopies and (B) radiological procedures per patient per year, 1 y before and 5 y after the index date for cases in Constant Care and their matched controls receiving standard care for the 2 cohorts.

In Cohort 1, cases underwent significantly more endoscopies in Years −1 and 2 (Figure 4). In Cohort 2, cases underwent more endoscopies than controls throughout the study period, but this difference did not reach statistical significance in Years 4 and 5.

The significantly greater number of radiological procedures in cases before the index year (*p* = 0.001) in both cohorts decreased, while remaining unchanged in controls, in Years 1, 4, and 5 for Cohort 1 and in Year 5 for Cohort 2; however, these differences over time were not statistically significant (Figure 4A and B).

In Cohort 1, the mean number of IBD-related surgeries in cases enrolled in CC was 0 before the index year and throughout all follow-up years, except for the index year, where it increased to 0.003, and it was lower throughout all years compared to controls receiving sCare. In Cohort 2, the mean number of IBD-related surgeries fell after inclusion in CC but increased in Years 1–5, overtaking the number of IBD-related surgeries among controls after Year 2 (Table 3).

**Table 3:** Mean number of IBD-related surgeries in Cohorts 1 and 2.

	Cases enrolled in CC		Controls receiving sCare	
Year	Mean	95% CI	Mean	95% CI
Number of surgeries in Cohort 1				
−1	0.000	(0.00–0.00)	0.005	(0.00–0.01)
0	0.003	(0.00–0.01)	0.005	(0.00–0.01)
1	0.000	(0.00–0.00)	0.002	(0.00–0.01)
2	0.000	(0.00–0.00)	0.002	(0.00–0.01)
3	0.000	(−0.01 to 0.01)	0.005	(0.00–0.01)
4	0.000	(−0.01 to 0.01)	0.000	(−0.01 to 0.01)

Number of surgeries in Cohort 2				
−1	0.023	(0.01–0.03)	0.020	(0.01–0.03)
0	0.005	(−0.01 to 0.01)	0.022	(0.01–0.03)
1	0.009	(0.00–0.02)	0.010	(0.00–0.02)
2	0.018	(0.01–0.03)	0.002	(−0.01 to 0.01)
3	0.020	(0.00–0.04)	0.004	(−0.02 to 0.02)
4	0.045	(0.01–0.08)	0.000	(−0.03 to 0.03)

Abbreviations: CC, Constant Care; CI, confidence interval; IBD, inflammatory bowel disease; sCare, standard care.

### 3.4. Indirect costs

**Figure 5:**
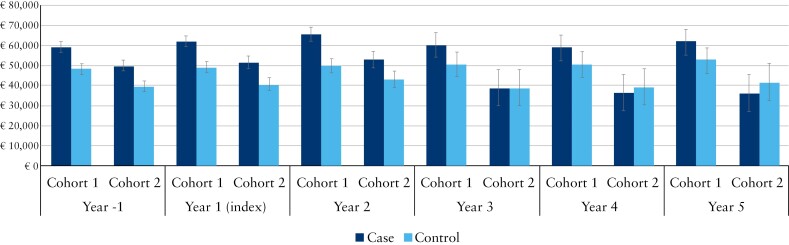
Mean total earnings per patient per year, from 1 y before, until 5 y after, the index date for cases enrolled in Constant Care and their matched controls receiving standard care for Cohorts 1 and 2. Abbreviation: CI, confidence interval.

Data for the mean earnings per patient per year in Cohorts 1 and 2 are shown in Figure 5. In Cohort 1, cases brought in significantly higher total earnings compared to controls throughout the study period, except in Year 4, where the difference was nonsignificant. In Cohort 2, cases also brought in significantly higher total earnings compared to controls until Year 3, at which point the difference became statistically nonsignificant; however, cases tended to bring in lower earnings from Year 3 onward.

In Cohort 1, cases had received fewer public transfers throughout all years except at Year 4, where cases received higher public transfers compared to controls in sCare. Overall, early retirement rates were lower in cases compared to controls. Data are shown in Figure 1 and Supplementary Figure 1 and its corresponding Supplementary Table 2.

In Cohort 2, cases started to receive greater public transfers from Year 4 onward compared to controls in sCare. Early retirement rates were lower in cases compared to controls in the first 4 years of implementation but tended to be higher by 1% at Year 5. Data are described in Figure 2 and Supplementary Table 3.

### 3.5. Patient-reported outcome measures within the telemedicine group

**Figure 6:**
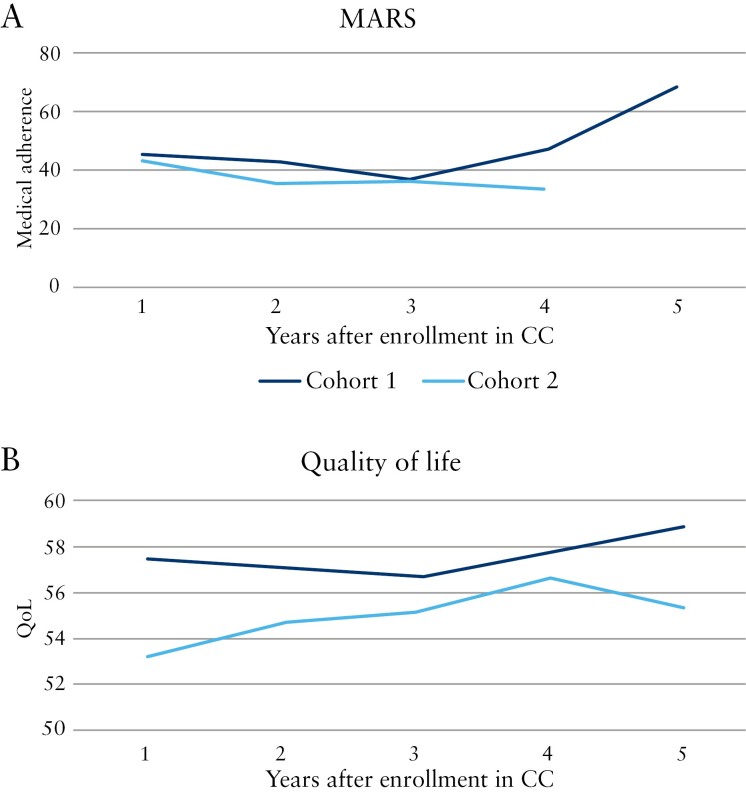
QoL and MARS scores from 1 to 5 y after the index date among cases enrolled in CC in Cohorts 1 and 2. Abbreviations: CC, Constant Care; MARS, Medication Adherence Rating Scale; QoL, quality of life.

QoL and MARS PRO data for patients enrolled in CC through 5 years of follow-up are presented in Figure 6; other PROs including SCCAI, HBI, WEB-DI, and fecal calprotectin are presented in Supplementary Figure 3. QoL was higher in Cohort 1 than in Cohort 2. Cohort 2 had decreasing QoL after Year 4. Based on SCCAI scores, disease activity among UC patients in Cohort 1 appeared to decrease after 3 years. According to HBI scores, disease activity among CD patients increased after Years 3 and 4 in Cohorts 1 and 2, respectively (however, this finding was based on a limited number of observations). WEB-DI scores decreased after Year 3 for both cohorts, especially for Cohort 1. Patients in Cohort 1 were more adherent to medication plans than patients in Cohort 2. Fecal calprotectin increased or remained stable around 200 mg/kg until Year 4, at which point it dropped markedly in Cohort 1 but remained stable under 250 mg/kg in Cohort 2.

## 4. Discussion

This is the first study to report the long-term follow-up costs of telemedicine in IBDs using a population-based sample of patients, where treatment choices reflect the preferences of both physicians and patients. We have previously explored the financial implications of CC across 3 years of follow-up, demonstrating that telemedicine can be a cost-effective alternative to sCare after the first 3 years of implementation.^21^ Our study spanned 5 years of follow-up and showed that PRO-based telemedicine was cost-effective in patients not receiving biologics when compared to sCare, but more expensive for patients receiving biologics. Furthermore, our assessment of PROs in the telemedicine group served as a secondary endpoint in our study and showed an improved QoL and adherence to medications, as well as less disability and better fecal markers, in patients not receiving biologics and mostly being treated with 5-ASA.

Overall, the number of IBD-related outpatient visits and hospital admissions in both cohorts was higher among cases than controls across 5 years of follow-up. This could be explained by healthcare contacts being easier to make for patients using telemedicine. A different explanation is that inexperience among nurses as to when to use the procedure code for eHealth consultations resulted in excessive visits being recorded. Additionally, during their first year, patients enrolled in CC were advised to register their symptoms every other week, or every 2 months if asymptomatic, and this could have biased the results, as patients received no such advice in Years 2–5.

However, the greater number of contacts with the healthcare system by patients in the telemedicine group is to be expected as part of the vigilant monitoring approach in the treat-to-target strategy for IBD.^39^ Telemedicine has been demonstrated to be an effective tool in controlling disease activity, as it allows for continuous monitoring and early detection of flare-ups and thus improves long-term patient outcomes.^1,21^ Furthermore, it can increase compliance and adherence, QoL, and self-initiated therapy with 5-ASA. Vigilant monitoring could also help explain the more frequent endoscopies and radiological procedures observed in cases than in controls for both cohorts of our study.

Yet another explanation for the higher number of IBD-related visits and admissions could be a more severe disease course among patients enrolled in the telemedicine group, and could also explain the increased number of patient contacts, in both cohorts, in the year before inclusion in the study. A previous randomized controlled trial^40^ demonstrated no superiority of telemedicine over sCare, with results that showed increased patient contacts and noninvasive diagnostic tests despite fewer hospitalizations. The trial concluded that the disease characteristics of participants at baseline were consistent with more severe IBD. However, our study did not match cases and controls on disease activity.

Despite the increase in the number of patient contacts in CC, telemedicine led to lower costs after Year 2 in Cohort 1, which constituted the majority of cases, compared to controls. This supports the argument that telemedicine improves disease outcomes and lowers IBD-related care costs, as previously shown in 2 randomized controlled trials^13,14^ of telemedicine in patients with IBDs who were followed up after 6 and 12 months. However, these studies found fewer phone calls, outpatient visits, and hospitalizations in addition to lower costs. In our study, a trend toward fewer radiological procedures after Year 4 was observed in Cohort 1, as well as zero surgeries in Years 1–4. This can be attributed to the involvement of patients in the management of their mild-to-moderate disease, that is, increased compliance^17^ and self-treating by adjusting the dose of 5-ASA, in accordance with the disease activity registered on CC.

In Cohort 2, expenditures for patients receiving biologics continued to be higher for cases than for controls between Years −1 and 5. Although a recent study showed a growing tendency to use biologics earlier on in IBD management,^41^ in Denmark, biologics are prescribed according to national guidelines and the instructions of the Danish Medicines Council.^28,42^ Data from our study showed an increase of 4% in the use of expensive biologics (Table 2), namely vedolizumab, ustekinumab, and tofacitinib, in addition to the less-expensive infliximab,^28^ after enrollment in CC. However, while the rate of bowel resections in these patients had declined in the index year and Year 2, the rate tended to increase markedly in the following 3 years compared to controls, which could be a result of the combined use of biologics and telemedicine, if careful monitoring was prolonging the time to surgery in IBD. In an earlier study, Qvist et al^43^ demonstrated that treatment with biologics does not significantly alter the cumulative surgical rates among patients with IBD. This finding could help to explain the higher expenses associated with biological therapy.

Using PROs, by including questionnaires for QoL, WEB-DI, and SCCAI/HBI, and measures of fecal calprotectin, CC can provide an overview of the total inflammatory burden of the disease and guide treat-to-target management of IBD. Within-group analysis in our study showed improvement in QoL and disability within the telemedicine group. A randomized trial from 4 centers in Holland evaluating eHealth (MyIBDCoach) for a year reported reduced outpatient visits and hospital admissions, as well as improved adherence to medications; however, the same study showed no difference in QoL between telemedicine and sCare groups.^12^ Our study showed increased disease activity after 3 years of follow-up, especially among patients with CD, which could partly be explained by careful monitoring prolonging the time to relapse. However, data about relapse rates in cases were not available and, furthermore, increasing HBI was observed in both cohorts.

Among the strengths of our study is its use of population-based register data retrieved through Statistics Denmark, from which a matched control group of IBD patients from all 5 regions in Denmark could be assembled. The Danish NPR is among the most comprehensive healthcare registries in the world.^44^ Another strength is the large sample size of cases enrolled in CC and controls receiving sCare, which allowed us to match them 1:5, respectively. Moreover, we were able to match cases and controls according to the type of biologics these patients received.

The study has several limitations. Variable registration techniques among different hospitals cannot be ruled out, which could partly explain the lower costs for sCare. The NPR does not indicate whether consultations are with a doctor or nurse. Neither telephone nor in-person consultations have unique codes in the national registry. The study did not estimate whether in-person visits decreased after enrollment in the telemedicine solution. Another limitation was that CC could not differentiate between patients that did not use the CC system any longer or who were running an indolent course and thus not needing CC validation. Another limitation was the fact that clinical and endoscopic disease activities are not recorded in the nationwide registries and hence were not available for the analysis. Furthermore, lack of malignancy rates was another limitation, which could have influenced the costs in patients with IBD. The omission of demographic and socioeconomic factors was a limitation in assessing the actual economic status of both cases and controls, which could have exerted a certain effect on indirect costs. Finally, the study is limited by an absence of data about transportation costs and waiting times.

## 5. Conclusion and future perspectives

Telemedicine promises a holistic, efficient, and cost-effective approach to treating IBDs, especially in comparison to sCare. Efforts should be made to standardize outpatient clinic follow-up policies, as well as to unify registration procedures for IBD patients. Ideally, randomized controlled trials will be carried out.

It is essential for healthcare providers to carefully assess which patients are the ideal candidates for telemedicine follow-up addressing the growing number of patients with IBDs, the increasing use of biologics, and the shortage of healthcare providers.

Patients in remission or those with mild-to-moderate disease activity may particularly benefit from telemedicine. These patients may not require frequent in-person visits with their gastroenterologist; however, this approach still ensures continuity of care and early detection of disease flare-up.

Also, telemedicine offers patients with moderate-to-severe disease the opportunity for self-monitoring with the use of home fecal calprotectin test, as they can easily track their disease activity and report any changes for timely intervention. In addition, pregnant individuals with IBDs can benefit from telemedicine follow-up as well. Telemedicine allows easy access to healthcare providers, enabling timely interventions and personalized care for this vulnerable population.

However, healthcare providers must also consider the individual needs and preferences of each patient when determining the most appropriate mode of care delivery. Nurses can play a crucial role in monitoring these patients closely and providing education and support. Therefore, it is essential for nurses to be well prepared and equipped with the necessary skills to facilitate telemedicine and effectively enroll patients in the system.

While the widespread use of digital health technology allows patients more direct and quicker access to health care professionals, dedicated time for technology use by health care professionals to respond to patient queries and formulate plans equally needs to be factored in. A restructuring of the clinical team job planning is likely going to be required, as technology becomes more and more an integral part of daily care.

## Supplementary Material

jjae120_suppl_Supplementary_Material

## Data Availability

Data cannot be shared for ethical/privacy reasons.
