# Supplementary material for: The Costs of Home Monitoring by Telemedicine vs Standard Care for Inflammatory Bowel Diseases—A Danish Register-Based, 5-Year Follow-up Study
Source: J Crohns Colitis. 2024 Aug 7;19(2):jjae120. doi: 10.1093/ecco-jcc/jjae120 (PMC11836881; doi:10.1093/ecco-jcc/jjae120)
Supplement: jjae120_suppl_Supplementary_Material [file jjae120_suppl_supplementary_material.docx]

**Supplementary material:**

| Procedure | Procedure code |
| --- | --- |
| Total colectomy | KJFH |
| Resections and excisions of the rectum | KJGB |
| Endoscopic procedures:  Gastroscopy  Gastroscopy with biopsy  Coloscopy  Coloscopy with biopsy  Flexible sigmoidoscopy  Flexible sigmoidoscopy with biopsy | KUJD02  KUJD05  KUJF32  KUJF35  KUJF42  KUJF45 |
| Radiological procedures:  Computer tomography – CT-scanning:  CT scan of the abdomen  CT scan of the upper abdomen  CT scan of the lower abdomen  Magnetic resonance imaging – MRI:  MR scan of the upper abdomen  MR scan of the lower abdomen  MR scan of the gastrointestinal tract | UXCD00  UXCD10  UXCD15  UXMD10  UXMD15  UXMD25 |
| Biologicals:  Infliximab  Adalimumab  Golimumab  Vedolizumab  Ustekinumab  Tofacitinib | BOHJ18A1  BOHJ18A3  BOHJ18A4  BOHJ18A4  BOHJ18B3  BOHJ28D |

Table 1: Procedure codes

**Results of indirect costs in cohort 1:**


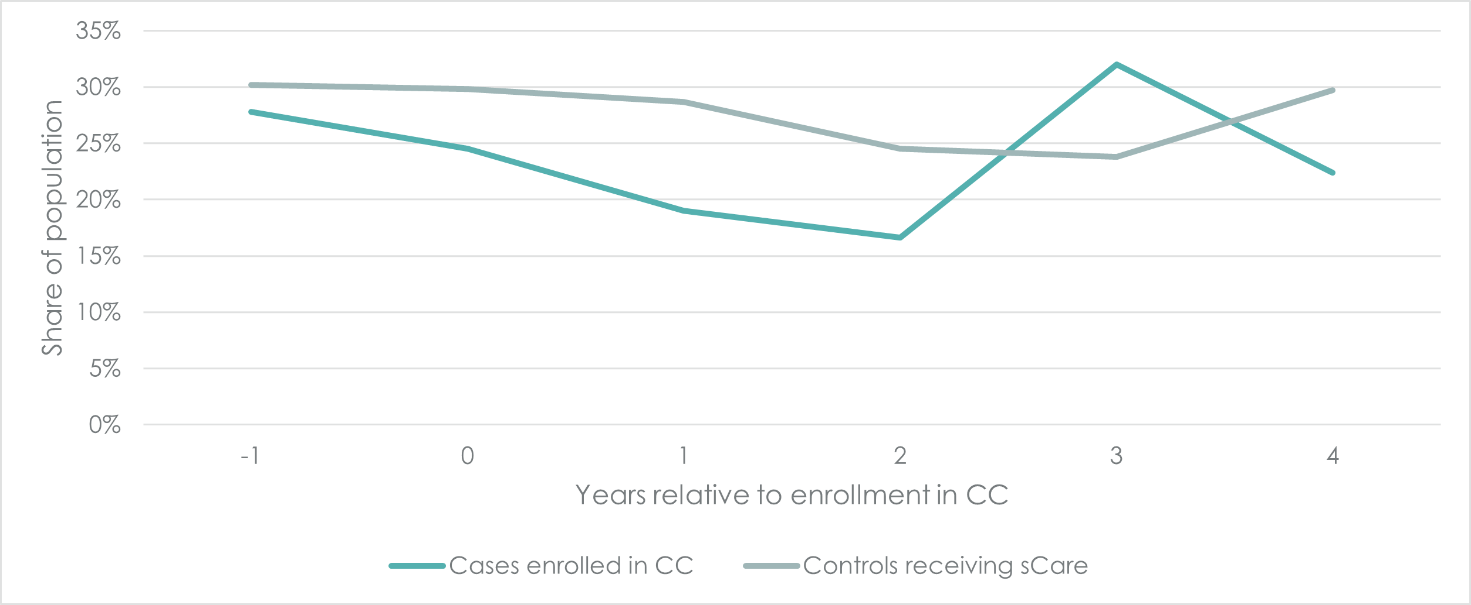


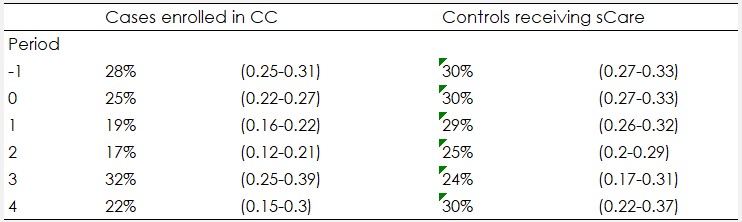


Figure 1: Share of population receiving public transfer payments per year, one year before and five years after the index date for cases in cohort 1 (not receiving biologics) in Constant Care and their matched controls receiving standard care.


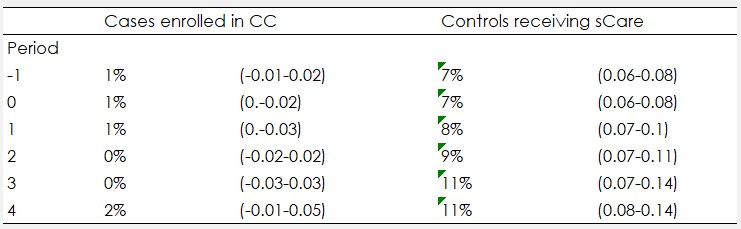


Table 2: Share of population on early retirement with one year before and five years after the index date for cases in cohort 1 (not receiving biologics) in Constant Care and their matched controls receiving standard care.

**Results of indirect costs in cohort 2:**

**
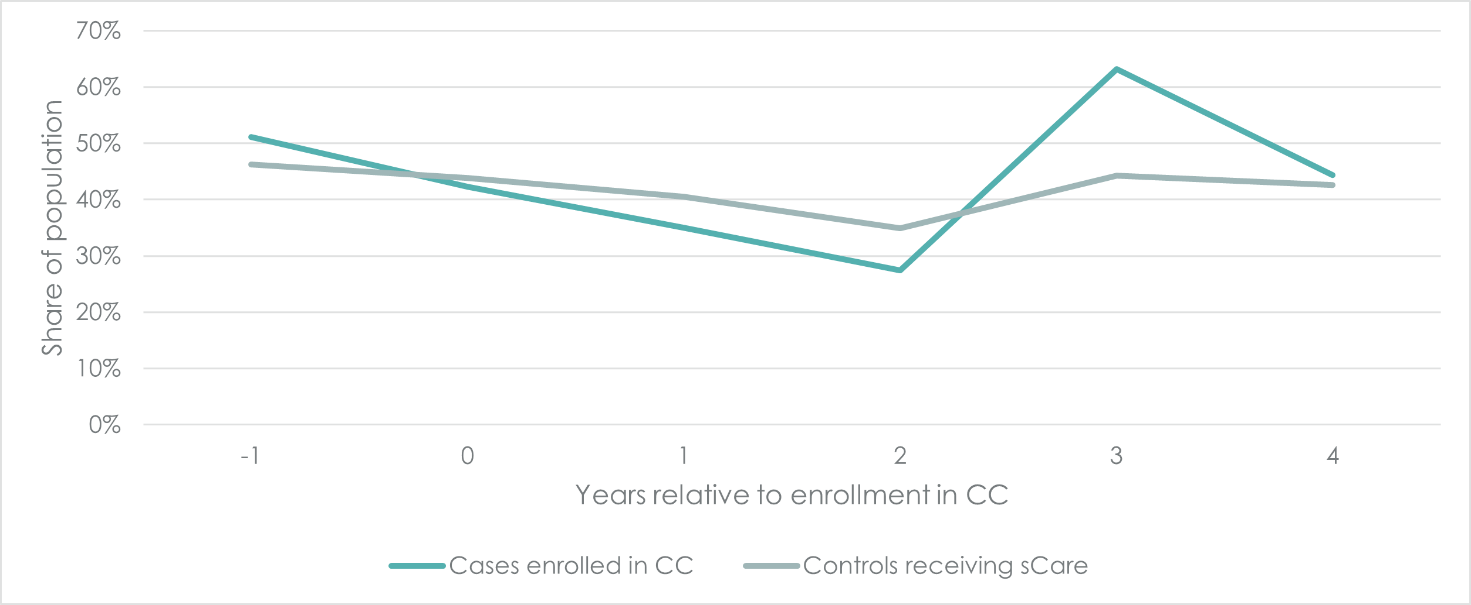
**


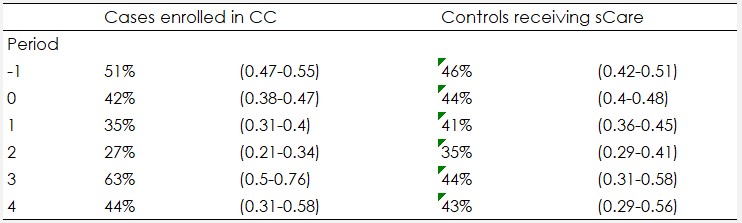


Figure 2: Share of population receiving public transfer payments per year, one year before and five years after the index date for cases in cohort 2 (receiving biologics) in Constant Care and their matched controls receiving standard care.


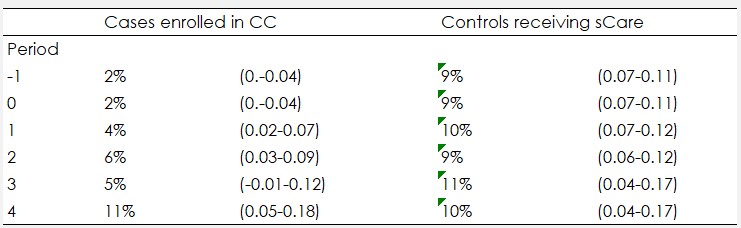


Table 3: Share of population on early retirement with one year before and five years after the index date for cases in cohort 2 (receiving biologics) in Constant Care and their matched controls receiving standard care.

**Patient-reported outcome (PRO) measures within the telemedicine group:**

**
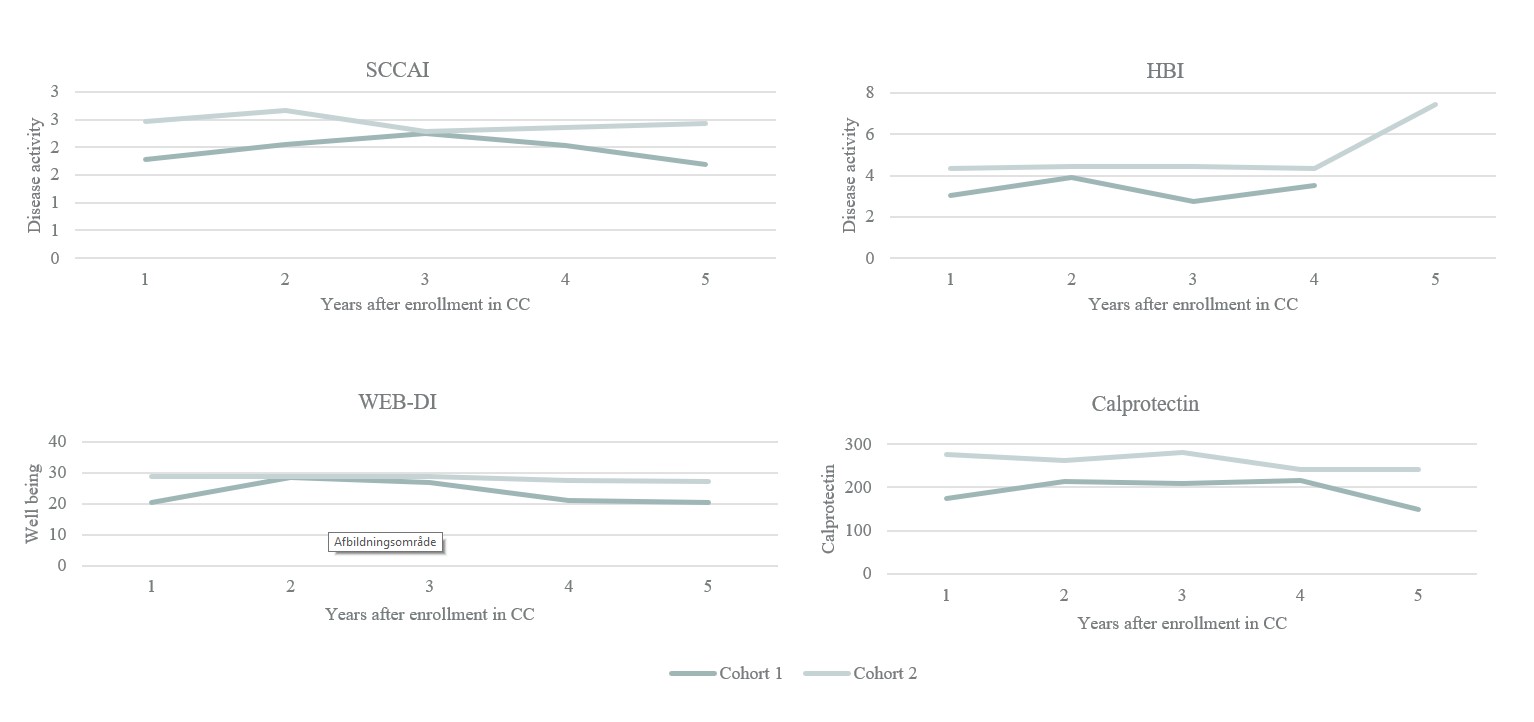
**

Figure 3: Simple Clinical Colitis Activity Index (SCCAI), Harvey-Bradshaw Index (HBI), web disability index (WEB-DI), and faecal calprotectin from one to five years after the index date among cases enrolled in Constant Care in cohorts 1 and 2
